# Relationship between Attention to Body Shape, Social Physique Anxiety, and Personal Characteristics of Brazilians: A Structural Equation Model

**DOI:** 10.3390/ijerph192214802

**Published:** 2022-11-10

**Authors:** Wanderson Roberto da Silva, Patrícia Angélica Teixeira, João Marôco, Eric Batista Ferreira, Micaela Aparecida Teodoro, Juliana Alvares Duarte Bonini Campos

**Affiliations:** 1 Graduate Program in Food, Nutrition and Food Engineering, São Paulo State University (UNESP), Araraquara 14800-903, São Paulo, Brazil; 2 Graduate Program in Nutrition and Longevity, Federal University of Alfenas (UNIFAL-MG), Alfenas 14800-903, Minas Gerais, Brazil; 3 William James Center for Research (WJCR), University Institute of Psychological, Social and Life Sciences (ISPA), 1149-041 Lisboa, Portugal; 4 Statistics Department, Federal University of Alfenas (UNIFAL), Alfenas 37130-001, Minas Gerais, Brazil

**Keywords:** body image, attention, shape, social, physique anxiety, personal characteristic, body composition, eating disorder, structural equation model

## Abstract

People can develop eating disorders due to excessive body image concerns. The primary objective of this study was to examine the relationship between attention to body shape, social physique anxiety, and personal characteristics in a sample of Brazilians. The secondary objective was to evaluate the correlation of the constructs with the participants’ body composition. First, 1795 individuals (70% female; *M*_age_ = 25.5 ± 6.6 years) completed the Attention to Body Shape Scale, the Social Physique Anxiety Scale, and a sociodemographic questionnaire. Then, 286 participants (58% female; *M*_age_ = 25.3 ± 5.7 years) underwent a bioimpedance exam to identify body composition. Structural equation modeling was used to estimate the relationship between the variables. The greater the attention to body shape, the greater the expectations of negative physical evaluation and the less comfort with physical presentation. Younger age, female gender, consumption of supplements/substances for body change, restrictive diets, physical inactivity, poor self-assessment of food quality, and overweight/obesity were related to negative body concerns. An expectation of negative physical evaluation was positively correlated with body fat and negatively with muscle mass. Comfort with physical presentation was negatively correlated with fat and positively with muscle. These results can support preventive strategies aimed at reducing eating disorders resulting from body image concerns.

## 1. Introduction

Body image is a field of interest for scientists and clinicians who seek to understand the breadth of this concept and its relationship with different contexts in people’s lives, such as eating behavior (e.g., emotional eating) and mental health (e.g., eating disorders). Investigating body image requires an insight into the complexity of this concept, which currently represents the entire experience that an individual can have, encompassing perceptions, beliefs, thoughts, feelings, and actions related to the body [1]. In view of this definition, the study of body image is challenging. Therefore, research has focused on the evaluation of one or more component aspects of this concept [2]. This study was developed with a focus on cognitive (attention to body shape) and affective (social physique anxiety) aspects of the attitudinal dimension of body image.

Attention can be understood as a selective cognitive processing where the individual intentionally focuses on something specific or based on internal and/or external stimuli. In this process, the person is consumed by some information while neglecting others, and this occurs individually and may vary according to the intensity of each stimulus [3]. In the context of body image, when individuals turn their attention to their physique, they can experience feelings of adequacy, but also of inadequacy, which, depending on the intensity, can trigger mental disorders such as anorexia or bulimia nervosa [4,5]. Therefore, assessing the degree of attention to the body becomes relevant to identify a dysfunctional cognitive process.

Anxiety is an adaptive human process represented by the anticipation of a future event that can cause, in some individuals, feelings of discomfort [6]. When an individual experiences anxiety in social environments that causes an emotional response, such as severe or intense fear that causes suffering with a negative affectivity, it can be considered a symptom of mental disorder [7]. Social anxiety can occur for different reasons, for example, insecurity with physical presentation in social environments can lead to feelings of inadequacy [7,8]. This process is called social physique anxiety and was introduced by American researchers [9] to represent an individual’s feelings when their appearance is evaluated by others in real or hypothetical situations.

According to Compton [3], cognitive and affective aspects are related, as both deal with information processing priorities. In this way, when individuals focus their attention on body regions that they consider unattractive, they may be uncomfortable with their physical appearance causing problems, such as body dissatisfaction [10], disordered eating [7], and anxiety [11]. According to Pawijit et al. [12], self-focused thinking about the body and the concern about being negatively evaluated can promote social physique anxiety. Thus, investigating the relationship between attention to body shape and social physique anxiety is relevant to guide strategies that reduce disorders arising from negative attitudes towards body image.

In addition, previous studies have shown that people’s personal characteristics can contribute to body image concerns and consequently negative mental health. For example, younger people and women have greater concerns about their own body image and are considered vulnerable groups [13,14]. Restrictive diets [15,16], less participation in physical activities [17], and the consumption of fitness supplements (e.g., protein and caffeine products) and pharmacological substances (e.g., anabolic steroids and diuretics) for body change [18,19] have been associated with excessive body image concern. Furthermore, individuals with higher levels of body fat and higher body mass index (BMI) are also more concerned about their body [20,21]. Therefore, studying the relationship of personal characteristics with the degree of attention to body shape and social physical anxiety is relevant to identify which groups need special care. 

In light of the review above, the primary objective of the study was to evaluate the influence of attention to body shape on social physique anxiety and of these constructs on personal characteristics of Brazilian individuals. As a secondary objective, the correlation of the constructs with the participants’ body composition was investigated. We used validated tools to assess the constructs and hypothesized that the greater the attention to body shape, the worse the social physical anxiety outcomes. In addition, we hypothesized that specific characteristics (e.g., younger age, female gender, being on a restrictive diet, and having more body fat) could impact negative outcomes related to body image.

## 2. Materials and Methods

### 2.1. Study Design and Participants

**Figure 1:**
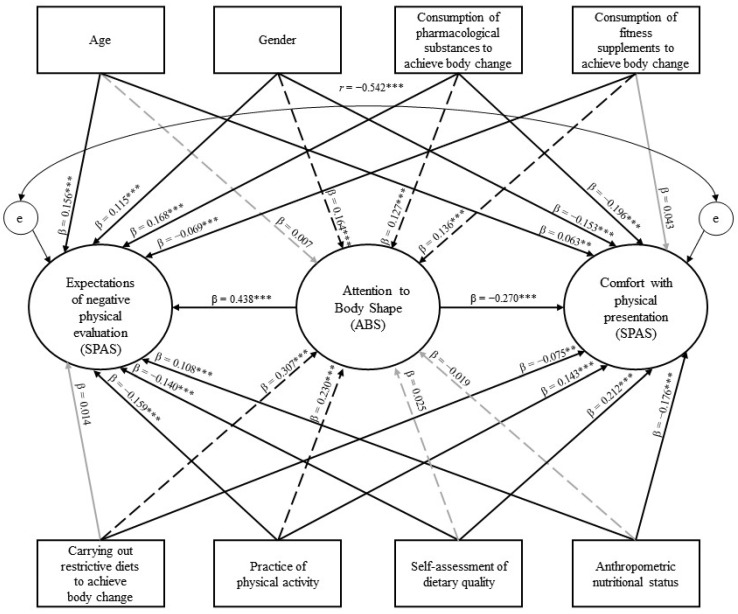
Hypothetically causal structural model designed to verify the relationship between the study variables. Note. *** *p* < 0.001, ** *p* < 0.01. Circles represent latent variables and rectangles represent manifest variables. Arrows: black indicates significant paths; grey indicates non-significant paths; dotted lines indicate mediation paths (confirmed by Sobel’s test). The final model is composed of all significant paths in black color. β: estimate of the path, e: residue, r: correlation coefficient, ABS: Attention to Body Shape Scale, SPAS: Social Physique Anxiety Scale.

This cross-sectional study was conducted in two stages. In the first stage, the calculation of the minimum sample size considered the need for 15 respondents for each parameter of the structural model to be tested [22]. The relationships between the variables came from evidence from the literature—as presented in the Introduction—and the model included 63 parameters (see Figure 1: 24 paths from manifest independent variables to latent dependent variables; 2 paths between latent variables; 25 parameters from the factor structure of the Social Physique Anxiety Scale [i.e., 12 items + 12 item residues + 1 correlation between factors]; and 12 parameters from the factor structure of the Attention to Body Shape Scale [i.e., 6 items + 6 item residues]). Thus, with 63 parameters and a loss rate of 20%, the calculated sample size was 1182 individuals. Brazilians of both genders (i.e., female and male) age 18 years and over were invited to participate in the study. The exclusion criteria were pregnancy, lactation, blindness, and people who reported having been diagnosed with a mental disorder in the last 12 months. To screen for inclusion in the definitive study, a questionnaire with the exclusion criteria was applied to each participant.

In the second stage, the calculation of the minimum sample size considered a correlation coefficient between the variables of 0.50, α = 5%, and β = 20%, which resulted in 80 individuals [22]. We decided to include at least 80 participants of each gender who were randomly selected from the sample of the first stage.

### 2.2. Study Variables and Instruments

Participants’ Personal Characteristics. The participants self-reported information about their gender, age, marital status, weight, height, consumption of fitness supplements and pharmacological substances to achieve body change, carrying out restrictive diets to achieve body change, physical activity, and the quality of their diet. Self-reported weight and height were used to define anthropometric nutritional status. Attention to body shape and social physique anxiety were investigated using the psychometric instruments.

Attention to Body Shape Scale (ABS). This scale was developed with seven items and five Likert response options, ranging from definitely disagree to definitely agree, to assess attention to body shape [23]. As reported in a previous study [5], excluding item three (“*I am not self-conscious about my body shape*”), the ABS showed good factorial validity and reliability (for review, see Brown [24]), which also occurred in the present study (total sample: Root Mean Square Error of Approximation [RMSEA] = 0.07 and 90% confidence interval [CI90%] = 0.06–0.09, Comparative Fit Index [CFI] = 0.99, Tucker-Lewis Index [TLI] = 0.98, omega coefficient [ω] = 0.86, alpha ordinal coefficient [α] = 0.85; female sample: RMSEA = 0.06 [CI90% = 0.05–0.08], CFI = 0.99, TLI = 0.98, ω = 0.85, α = 0.85; male sample: RMSEA = 0.08 [CI90% = 0.06–0.10], CFI = 0.99, TLI = 0.98, ω = 0.87, α = 0.86). Therefore, the ASB 6-item one-factor model was used.

Social Physique Anxiety Scale (SPAS). This scale was developed with 12 items and five Likert-type response options, ranging from not at all to extremely characteristic, to assess social physique anxiety [9]. A two-factor model [25,26] of the scale was used, which makes it possible to evaluate “expectations of negative physical evaluation (F1)” and “comfort with physical presentation (F2)”. This model showed good factorial validity and reliability in the present study (total sample: RMSEA = 0.08 [CI90% = 0.08–0.09], CFI = 0.97, TLI = 0.96, ω_F1_ = 0.81, ω_F2_ = 0.90, α_F1_ = 0.80, α_F2_ = 0.90; female sample: RMSEA = 0.09 [CI90% = 0.08–0.10], CFI = 0.96, TLI = 0.96, ω_F1_ = 0.90, ω_F2_ = 0.81, α_F1_ = 0.80, α_F2_ = 0.89; male sample: RMSEA = 0.08 [CI90% = 0.06–0.09], CFI = 0.98, TLI = 0.97, ω_F1_ = 0.79, ω_F2_ = 0.91, α_F1_ = 0.78, α_F2_ = 0.90).

To verify the plausibility of developing a single structural model for both genders, multigroup analysis (for reviews, see Putnick and Bornstein [27] and Chen [28]) was performed to estimate the invariance (i.e., equivalent across different groups) of the factor model for each scale (i.e., ABS and SPAS). The CFI difference statistic (ΔCFI) was used to compare factor loadings, thresholds, and residues across groups. The CFI values of the configural, metric, and scalar models were compared two by two, and the factor model for each scale was considered invariant if the decrease in CFI was less than −0.01 [28]. As the invariance was found for both ABS (ΔCFI: −0.002–0.001) and SPAS (ΔCFI: −0.004–0.002), a single structural model was built for both genders.

### 2.3. Procedures

Initially, the research was released at the university where the study was conducted to students, technical-administrative employees, and professors through personal invitations and the internet (e.g., e-mail, Instagram and Facebook). Ethical approval was given by the university where the study was developed. Two researchers were trained for data collection. Individuals who were interested in the research and were eligible received guidance on the purpose of the study and the form of voluntary participation (i.e., without financial or academic incentives). Before filling in the data, the participants signed the Free and Informed Consent Form and were informed that the research would be developed in two stages of data collection and if they were invited to participate in the second collection they could accept or refuse the invitation. To enable the random drawing of participants in the second stage, a numerical code was assigned to each questionnaire, and on the first page, their name and telephone or e-mail were requested. The participants filled out questions formulated to characterize the sample along with the Portuguese versions of the ABS [5] and SPAS [29] using paper and pen. Data collection was performed in a room reserved for this purpose located at the university. The presence of the researcher in this room was only to distribute or collect the questionnaire to maintain the volunteers’ privacy while completing them. After completing the questionnaires, each individual was asked to tell others about the research, thus adopting a snowball sampling process (for review, see Johnson [30]). The period of data collection for the first stage was from March 2018 to July 2019 and the second stage was from August to December 2019. Importantly, two validation studies related to the present study have been published [5,26].

### 2.4. Structural Model (First Stage)

To verify the relationship between attention to body shape, social physique anxiety, and participants’ personal characteristics, a structural model was built and tested using the structural equation modeling technique. The hypothetical pathways (β) were tested stepwise. First, we verified the influence of attention to body shape (independent variable evaluated from ABS) on expectations of negative physical evaluation (dependent variable evaluated from SPAS), and on the comfort with physical presentation (dependent variable evaluated from SPAS). Then, the participants’ personal characteristics were dichotomized (gender [0: male, 1: female]; age [0: <30 years old, 1: ≥30 years old], anthropometric nutritional status [0: overweight/obesity absent, 1: overweight/obesity present], consumption of fitness supplements and of pharmacological substances to achieve body change [0: no, 1: yes], carrying out restrictive diets to achieve body change [0: no, 1: yes], self-assessment of the quality of their diet [0: poor, 1: good]; practice of physical activity [0: no, 1: yes]) and inserted into the model as independent variables to assess their influence on the attention to body shape, on expectations of negative physical evaluation, and on the comfort with physical presentation (dependent variables) (see Figure 1).

For analyses, we used the weighted least squares means and variance adjusted (WLSMV) estimation and the z test. First, the quality of the fit of the structural model to the data was verified using three goodness of fit indices (acceptable values for good fit: RMSEA ≤ 0.10, CFI > 0.90, and TLI > 0.90) [22,31]. Then, each β was analyzed and compared to the critical ratios of z. A significance level of 5% was used. The R (version 3.6.2, CRAN Task Views) through packages lavaan [32], semTools [33], and psych [34] was used for data analysis.

### 2.5. Participants and Procedures (Second Stage)

In the second stage, the individuals were invited at random from the sample in first stage. They returned to the university to perform the body composition assessment and again filled out the questionnaire to characterize the sample and the scales items (ABS and SPAS). A properly trained nutritionist performed the data collection. When invited to the second stage, this professional told the individuals that they would perform a bioimpedance exam and that on the day of collection they should wear light clothing and fast for 2 h. In addition, they should not drink alcohol or caffeine, practice physical activity for 24 h prior to the assessment, or be menstruating. All participants provided written consent to participate in the study.

After each individual filled in the data using paper and a pen, their height was measured with a 210 cm compact stadiometer that was fastened to a smooth surface without a footer. The measurement was taken with the individual under the equipment, barefoot, without head adornments (e.g., headband or cap), standing erect, with feet and legs parallel, arms relaxed, head aligned in the Frankfurt plane, and with the posterior parts of the body in contact with the surface. Then, weight and body composition measures (i.e., body fat mass, skeletal muscle mass, BMI, body fat percentage, and visceral fat level) were obtained using high-frequency tetrapolar equipment (Biospace, InBody 570). To perform the bioimpedance test, the individual was asked to place bare feet and hands at the points indicated by the equipment. Then, the participant’s age, gender, and height were entered into the equipment and the test was performed.

For data analysis, the mean scores for attention to body shape (ABS), expectations of negative physical evaluation (SPAS), and comfort with physical presentation (SPAS) were calculated and correlated with body composition variables. Pearson’s correlation coefficient was used with a significance level of 5%. SPSS Statistics for Windows (version 28; IBM SPSS, Chicago, IL, USA) was used for the data analysis.

## 3. Results

In the first stage, the 1795 participants (70% female) had an average age of 25.5 ± 6.6 years. The average BMI of the participants was 24.4 ± 7.3 kg/m^2^. Detailed information about this sample is provided in Table 1.

Most individuals were single, reported never having consumed fitness supplements or pharmacological substances for body change, practiced physical activity, have already tried a restricted diet, assessed the quality of their diet as normal, and were classified according to BMI with normal weight.

Figure 1 presents the structural model evaluated and the standardized β obtained. Initially, we found that the construct evaluated by ABS significantly influenced (*p* < 0.001) the SPAS factors, indicating that the greater the attention to body shape, the greater the expectations of negative physical evaluation and the lower the comfort with physical presentation of the participants. Then, we found that all independent variables—selected from the characterization of the sample—contributed significantly (*p* < 0.01) to one or more of the latent concepts investigated.

Higher levels of attention to body shape were exhibited by female participants and those who reported consuming fitness supplements and pharmacological substances for body change, used restrictive diets, and practiced physical activity. Higher levels of expectations of negative physical evaluation were found in younger individuals, female participants, those who reported consuming pharmacological substances for body change and did not consume fitness supplements, those who did not practice physical activity, those who assessed the quality of their food as poor, and those who were classified as overweight/obese.

Higher levels of comfort with physical presentation were presented by older individuals and male participants, as well as those who reported not consuming pharmacological substances for body change or eating restrictive diets, practiced physical activity, rated the quality of their food as good, and those classified as overweight/obesity absent. After excluding non-significant pathways, the final model presented good fit to the data (RMSEA = 0.06 [CI90% 0.06–0.06], CFI = 0.93, TLI = 0.92, see Figure 1).

In the second stage, 286 individuals (58% female) participated, who had a mean age of 25.3 ± 5.7 years and a mean BMI of 24.0 ± 4.0 kg/m^2^. More details about the subsample are presented in Table 1. Most people reported being single, never having consumed pharmacological substances for body change, sometimes consuming fitness supplements, practicing physical activity, had already undertaken a restrictive diet, assessed the quality of their food as normal, and were classified according to BMI with normal weight. Table 2 contains the estimated correlation between the body composition components and the mean scores of the scale factors.

The attention to body shape did not show significant correlations with body composition. There were positive and significant correlations (*p* < 0.05) between expectations of negative physical evaluation and body fat mass, body fat percentage, visceral fat level, and attention to body shape. There were negative and significant correlations of comfort with physical presentation with body fat mass, BMI, body fat percentage, visceral fat level, attention to body shape, and expectations of negative physical evaluation. The significant correlation between skeletal muscle mass and expectations of negative physical evaluation was negative, while comfort with physical presentation was positive.

## 4. Discussion

The findings of the present study expand the body of evidence about the existence of a significant relationship between attention to body shape and social physique anxiety, and the association of these constructs with participants’ personal characteristics. We also verified a significant correlation between the participants’ body composition and components of the social physique anxiety. As far as we know, these specific relationships, evaluated simultaneously, had not yet been studied in Brazilian samples, which highlights the contribution of this study that may be useful for future protocols that seek to understand the relationship of individuals with their body image. Our results can also help public and clinical strategies aimed at reducing eating disorders, such as anorexia and bulimia nervosa, which are commonly related to negative body image.

We found that the greater attention to body shape, the greater the expectations of negative physical evaluation and the less comfort with physical presentation. This corroborates the literature [10,11,12,35], which points out that selective attention to the body—especially to the parts considered by the individual as unattractive—can be important for the development of feelings of inadequacy that can trigger social anxiety disorders, depression, and eating disorders, among others. Thus, teaching people to value their body, reducing social comparisons based on aesthetic standards, and encouraging engagement in activities that stimulate balance and pleasure can help to minimize dysfunctional behaviors that compromise health and well-being.

In our study, females paid more attention to body shape and had higher levels of social physique anxiety. In addition, younger people had higher expectations of negative physical evaluation and lower comfort with physical presentation. These findings are in line with the literature [13,14], which supports the vulnerability of these groups regarding aesthetic issues. Developing strategies aimed at these groups is important; however, this is a challenging task in the contemporary world that encourages behaviors that favor obtaining an “ideal” body image, which is unrealistic for most of the population [36]. Thus, more assertive actions must be implemented, such as the transmission of images with different types of body shapes and sizes whose appearance is closer to the general population.

The participants who reported consuming fitness supplements and pharmacological substances were more attentive to body shape and experienced greater social physique anxiety. Previous research [18,19,37] found that the use of these products to modify parts of the body “considered inappropriate” is common; however, this strategy does not always promote a positive body image. According to Brunet et al. [38], social physique anxiety influences the drive for muscularity and thinness, which may explain the use of supplements and substances to achieve an “ideal” body image [39]. These behaviors are generally adopted by individuals who care more about the body, such as those in fitness settings [40]. Therefore, these people should be encouraged to seek qualified professionals who develop therapeutic plans that are not centered on the use of “fitness products”, but rather on the valuation of the individual characteristics of the body itself.

Physical activity promoted greater attention to body shape and comfort with physical presentation. On the other hand, the lack of physical activity promoted expectations of negative physical evaluation. These findings corroborate the literature [8,17,40], which indicate that people’s engagement in physical activities, exercises, or sports can improve their relationship with body image. However, these people generally continue to worry about their physical appearance, as they gradually seek to verify whether the activity or exercise performed is having the desired body effects, which may explain the greater attention to body shape. When these effects are achieved, the person can feel more comfortable and confident exposing their body in social environments. However, when the individual does not practice physical activity or even when they do but they are unable to achieve the desired results, feelings of inadequacy may occur [17]. Thus, qualified professionals should encourage physical activities as a way of valuing health and well-being and not just physical appearance. This is a challenge, as it must be done individually according to each person’s needs.

Practicing restrictive diets promoted greater attention to body shape and less comfort with physical presentation. Previous studies [15,16,41] have demonstrated that rigid eating behaviors that restrict calories and nutrients promote a range of clinical, physical, and psychological disorders, including those related to body image. People believe that restrictive diets are effective in reducing body weight and promoting additional improvements such as greater romantic opportunities, self-esteem, and body satisfaction. However, this behavior can cause more harm than good, as it stimulates the internalization of dysfunctional beliefs that can trigger thoughts and feelings of inadequacy, especially regarding the body, which explains our findings [41].

As for dietary consumption, our study found that participants who classified their diet as poor exhibited more compromised social physique anxiety outcomes. As far as we know, no study has investigated the relationship between these variables, which only allows us to speculate. It was noteworthy that 75.6% of the people who classified the quality of their food as poor presented overweight or obesity, which are generally associated with inadequate food and body dissatisfaction [14]. Greater social physique anxiety among individuals with BMI ≥ 25 kg/m^2^ was also found. This finding extends those of previous research [20,42,43] that highlight the need to develop more targeted programs for people classified as overweight/obese, in order to promote a more empathetic relationship with the body through self-care to reduce feelings of inadequacy. Trained professionals should teach people to have eating behaviors appropriate to their socio-cultural context and with a variety of nutritious foods, so that food is not a reinforcer of negative body image. In addition, public health campaigns to reduce the stigmatization of body weight must be implemented.

Although BMI from self-reported measurements is a viable predictor for determining anthropometric nutritional status [44], the literature encourages body composition to be assessed in a more accurate way, i.e., measuring fat and lean mass [45,46], as performed in the second stage of this study. We found that participants with higher levels of body fat and visceral fat had higher expectations of negative physical evaluation and lower comfort with physical presentation. An inverse relationship was observed with skeletal muscle mass. These data are in line with the literature [21,47,48,49] supporting that people with a higher BMI experience a more negative body image and, thus, require special attention.

The findings of this study also indicated that attention to body shape did not correlate significantly with body composition. We believe that this may have occurred because the “external body” (i.e., physical appearance “seen with the naked eye”) is more valued by individuals than the “internal body” (i.e., body composition) [50]. This may partially explain our findings, considering that individuals may determine that the shape of their waist is adequate and not pay attention to that part of the body, even though they have a greater than the recommended level of visceral fat. However, future studies should evaluate this relationship in other contexts to support, expand, or refute our results.

Like all studies, this one has limitations. First, the cross-sectional design does not permit confirmation of a cause-effect relationship between the variables. Nevertheless, cross-sectional studies help to identify characteristics to be included in experimental and longitudinal research. The second is related to the use of a non-probabilistic and predominantly female sample that makes it impossible to generalize the results to different contexts; however, we sought to minimize this limitation using an extended sample. Third, some questions used to investigate the characteristics of the sample can be considered limiting; thus, we suggest expanding this investigation in future studies.

## 5. Conclusions

Our study pointed out that the greater the attention to body shape, the higher the expectations of negative physical evaluation and the lower the comfort with physical presentation. We also found that younger age, female gender, consumption of fitness supplements and pharmacological substances for body change, restrictive diets, physical inactivity, poor self-assessment of food quality, and overweight/obesity were personal characteristics related to dysfunctional thoughts and feelings about the body image. Finally, we identified that body fat was associated with higher scores for social physique anxiety. Therefore, people with these characteristics need more attention from professionals who work to promote physical, mental, and social well-being.

## Figures and Tables

**Table 1:** Characterization of study participants considering the two stages of the evaluation.

	Stage 1 Sample (n = 1795) n (%)	Stage 2 Subsample (n = 286) n (%)
**Gender**		
Male	540 (30.0)	120 (42.0)
Female	1255 (70.0)	166 (58.0)
**Marital status**		
Single	1422 (79.2)	239 (83.6)
Married	330 (18.4)	43 (15.0)
Divorced	40 (2.2)	4 (1.4)
Widowed	3 (0.2)	-
**Have you ever consumed pharmacological substances to achieve body change?**		
Never	1003 (55.8)	135 (47.9)
Once in lifetime	193 (10.8)	29 (10.3)
Sometimes	501 (27.9)	97 (34.4)
Often	98 (5.5)	21 (7.4)
**Have you ever consumed fitness supplements to achieve body change?**		
Never	784 (43.7)	77 (27.3)
Once in lifetime	212 (11.8)	32 (11.3)
Sometimes	585 (32.6)	117 (41.5)
Often	214 (11.9)	56 (19.9)
**Do you practice physical activity?**		
Yes	1.025 (57.1)	200 (69.9)
No	770 (42.9)	86 (30.1)
**Have you ever been on a restrictive diet to achieve body change?**		
Never	551 (30.7)	66 (23.2)
Once in lifetime	257 (14.3)	41 (14.4)
Once in a while	602 (33.5)	96 (33.8)
Sometimes	253 (14.1)	58 (20.4)
Often	132 (7.4)	23 (8.2)
**How would you rate the quality of your eating?**		
Poor	150 (8.3)	24 (8.4)
Fair	468 (26.1)	57 (19.9)
Normal	622 (34.7)	98 (34.3)
Good	492 (27.4)	93 (32.5)
Excellent	63 (3.5)	14 (4.9)
Anthropometric nutritional status *		
Underweight	97 (5.4)	13 (4.5)
Normal weight	1072 (59.7)	184 (64.3)
Overweight	421 (23.5)	64 (22.4)
Obesity	205 (11.4)	25 (8.8)
**Body composition ^†^**	**M ± SD**	**M ± SD**
Body fat mass (kg)	-	18.9 ± 8.9
Skeletal muscle mass (kg)	-	27.8 ± 6.9
Body fat percentage (%)	-	27.1 ± 9.4
Visceral fat level	-	7.9 ± 4.5
**Score ^‡^**		
ABS	3.2 ± 1.0	3.4 ± 0.8
ENPE (SPAS)	2.7 ± 1.1	2.7 ± 1.1
CPP (SPAS)	2.7 ± 0.9	2.6 ± 0.8

Note. * The anthropometric nutritional status was classified based on the weight and height of the participants using self-reported measures for the total sample and measured by the researcher for the subsample. ^†^ The results were obtained across bioelectrical impedance analysis using the InBody 570. Body fat mass is the sum of subcutaneous fat, visceral fat, and fat surrounding muscles. Skeletal muscle mass is the amount of muscle attached to the bones. Body fat percentage is based on the muscle to fat ratio. Visceral fat level refers to an estimation of abdominal fat that is known to be closely related to cardiovascular diseases (<10 indicates low risk; 10 is 100 cm^2^ of visceral fat). ^‡^ Average calculated from participants’ responses to the items of each scale. M: mean, SD: standard deviation, ABS: Attention to Body Shape Scale, SPAS: Social Physique Anxiety Scale, ENPE: expectations of negative physical evaluation, CPP: comfort with physical presentation.

**Table 2:** Correlation matrix between mean scores of the constructs evaluated by the Attention to Body Shape Scale and Social Physique Anxiety Scale with the components of body composition.

	BFM	SMM	BMI	BFP	VFL	ABS	ENPE	CPP
BFM	1							
SMM	0.041	1						
BMI	0.842 ***	0.467 ***	1					
BFP	0.854 ***	−0.451 ***	0.517 ***	1				
VFL	0.981 ***	−0.013	0.801 ***	0.874 ***	1			
ABS	0.029	−0.019	0.090	0.057	0.035	1		
ENPE	0.259 ***	−0.218 ***	0.105	0.332 ***	0.262 ***	0.199 **	1	
CPP	−0.361 ***	0.179 **	−0.205 ***	−0.403 ***	−0.361 ***	−0.168 **	−0.692 ***	1

Note. *** *p* < 0.001, ** *p* < 0.01. BFM: body fat mass, SMM: skeletal muscle mass, BMI: body mass index, BFP: body fat percentage, VFL: visceral fat level, ABS: attention to body shape, ENPE: expectations of negative physical evaluation, CPP: comfort with physical presentation.

## Data Availability

The data presented in this study are available on request from the corresponding author. The data are not publicly available due to not obtaining consent from respondents to publish the data.
